# Phenol-Explorer 3.0: a major update of the Phenol-Explorer database to incorporate data on the effects of food processing on polyphenol content

**DOI:** 10.1093/database/bat070

**Published:** 2013-10-06

**Authors:** Joseph A. Rothwell, Jara Perez-Jimenez, Vanessa Neveu, Alexander Medina-Remón, Nouha M'Hiri, Paula García-Lobato, Claudine Manach, Craig Knox, Roman Eisner, David S. Wishart, Augustin Scalbert

**Affiliations:** ^1^International Agency for Research on Cancer (IARC), Nutrition and Metabolism Section, Biomarkers Group, 150 cours Albert Thomas, F-69372 Lyon Cedex 08, France, ^2^INRA, UMR1019, Unité Nutrition Humaine, CRNH Auvergne, F-63000 Clermont-Ferrand, France, ^3^Department of Biological Chemistry, Institute of Advanced Chemistry of Catalonia (IQAC-CSIC), 18-26 c/ Jordi Girona, 0834, Barcelona, Spain, ^4^Nutrition and Food Science Department, School of Pharmacy, Diagonal Campus, 27-31 Joan XXIII, 0828, University of Barcelona, Barcelona, Spain, ^5^CIBER: BC06/03 Fisiopatologia de la Obesidad y la Nutrición, CIBERobn. Instituto de Salud Carlos III, 4 c/ Sinesio Delgado, 28029 Madrid, Spain, ^6^In Siliflo Inc, ABT6V1Y2, Edmonton, Canada and ^7^Departments of Computing Science and Biological Sciences, University of Alberta, Edmonton AB T6G 2E8, Canada

## Abstract

Polyphenols are a major class of bioactive phytochemicals whose consumption may play a role in the prevention of a number of chronic diseases such as cardiovascular diseases, type II diabetes and cancers. Phenol-Explorer, launched in 2009, is the only freely available web-based database on the content of polyphenols in food and their *in vivo* metabolism and pharmacokinetics. Here we report the third release of the database (Phenol-Explorer 3.0), which adds data on the effects of food processing on polyphenol contents in foods. Data on >100 foods, covering 161 polyphenols or groups of polyphenols before and after processing, were collected from 129 peer-reviewed publications and entered into new tables linked to the existing relational design. The effect of processing on polyphenol content is expressed in the form of retention factor coefficients, or the proportion of a given polyphenol retained after processing, adjusted for change in water content. The result is the first database on the effects of food processing on polyphenol content and, following the model initially defined for Phenol-Explorer, all data may be traced back to original sources. The new update will allow polyphenol scientists to more accurately estimate polyphenol exposure from dietary surveys.

Database URL: http://www.phenol-explorer.eu

## Introduction

Human diets are rich in polyphenols, which are major plant secondary metabolites abundant in many plant foods (1). Western populations consume an estimated 1–2 g/day polyphenols, mainly from fruits, vegetables and beverages such as tea, coffee, wine and fruit juices (2, 3). Owing to the presence of reactive phenolic groups in their structure, polyphenols exert a range of biological activities, and various epidemiological studies and clinical trials have linked their intake with a reduced risk of chronic diseases, such as coronary heart disease, stroke, type II diabetes and some cancers (4–6). For this reason, they are now regarded as important components of a healthy diet, and are thought to be partly responsible for the health benefits of an increased fruit and vegetable consumption (7). Polyphenol bioactivity, for example, may explain the protective effects of tea against cardiovascular diseases (8, 9) or of coffee against type II diabetes (10). Consequently, scientific and commercial interest in polyphenols has grown dramatically in recent years, and a large volume of literature on their bioactivities, metabolism and health effects is available.

To understand the precise effects of polyphenols on health and disease, large epidemiological studies are needed, for which the most accurate possible assessment of polyphenol intake is required. Individual intakes are calculated largely from dietary surveys, where participants report their food intake over a prescribed period. One commonly used tool is the 24-h dietary recall, where all food and drink items consumed in the preceding 24 h are recorded. Food frequency questionnaires are less detailed but can be obtained from more subjects. However, few researchers have incorporated the effects of food processing into polyphenol intakes estimated by these methods. Many polyphenol-rich foods are subject to a range of processing steps before eventual consumption, whether for storage, preservation or cooking purposes. As is the case for other dietary bioactives, some processes lead to substantial polyphenol losses or to conversion between polyphenol forms (11), and often only polyphenol composition data for raw foods are available.

To accurately measure polyphenol intake in epidemiological studies, an additional coefficient, or *retention factor*, should be applied to the content value of each polyphenol in raw foods to account for processing (12). Retention factor is defined as

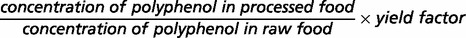

where

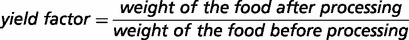

The yield factor takes into account change in weight of the food due to processing. In many cases, heat from processing causes loss of water, although some foods, such as rice, absorb water when boiled or steamed.

A retention factor for a given polyphenol is specific to both the process and the food. Published data on polyphenol retention factors are numerous, although scattered until now across a large number of publications and not easily available from purpose-built databases. Few retention factor databases have been developed for food nutrients; the United States Department of Agriculture (USDA) Table of Nutrient Retention Factors (13) and Bognar’s tables on retention factors of food constituents (14) contain data on vitamins, minerals and other micronutrients, but not polyphenols.

Since its launch in 2009, the first release of Phenol-Explorer (Phenol-Explorer 1.0) has aided estimations of polyphenol intake by generating an instant estimate of the polyphenol composition of a queried food, based on data from as many quality-assessed publications as possible (15). Similarly, it was possible to search for a food polyphenol, and retrieve all foods in which this polyphenol had been identified, along with average concentrations. A second update, launched in 2011 (Phenol-Explorer 2.0) (16), added data on the metabolism and pharmacokinetics of polyphenols, compiled from >200 dietary intervention studies performed with humans or experimental animals. As a comprehensive polyphenol knowledge base, the database has become a unique and essential tool for polyphenol scientists.

Although many studies have determined polyphenol losses from specific foods after processing, no method has hereto been available to calculate, collate and aggregate polyphenol retention factors. Here we report the third major release of Phenol-Explorer (Phenol-Explorer 3.0), which introduces data on retention factors for polyphenols in foods. Searches are made through the existing user-friendly and intuitive web interface, and retention factor data are linked to food composition data. In the same way as for composition data, as many similar data as possible from different publications were aggregated to give as reliable estimates as possible.

### Compilation of data

A systematic search was performed for literature on the effects of processing on the polyphenol contents of foods, published up until 1 April 2012. Searches were made in turn for foods from seven food groups (alcoholic beverages, cereals and cereal products, fruits and fruit products, nonalcoholic beverages, oils, seeds or vegetables), consistent with the ontology developed for the first release of Phenol-Explorer (15). Search terms for individual foods (e.g. ‘spinach’) were combined with those for specific polyphenols (‘ferulic acid’) or polyphenol classes (e.g. ‘flavonoid OR phenolic acid’) and some keywords related to quantitative information (e.g. ‘content OR determine* OR quantif*’) and food processing (e.g. ‘cook* OR process* OR fried OR frying OR boil* OR steam* OR stor*’). All searches were made using the Thomson Reuters Web of Knowledge Science Citation Index Expanded (1945–present). The publications retrieved were compiled and organized using Endnote (Thomson Reuters, New York, NY). In addition, publications used for the first release of Phenol-Explorer (polyphenol composition of foods only) were checked for relevant food processing data, and their reference lists checked for other relevant publications.

Titles and abstracts were screened and any non-relevant publications eliminated. Of the remainder, full-text articles were obtained for more detailed review. Articles were subsequently retained for data compilation or excluded according to several criteria. Studies needed to (i) be conducted on polyphenol-containing foods consumed by humans; (ii) investigate the effects of one or more processing methods on the polyphenol content of those foods; (iii) use extraction and analysis techniques that reliably quantified food polyphenols; (iv) give polyphenol contents, calculated by corresponding analytical methods, for the same foods before and after processing; (v) be published in English. In addition, some articles were excluded owing to a lack of clarity or essential information.

**Figure 1.**
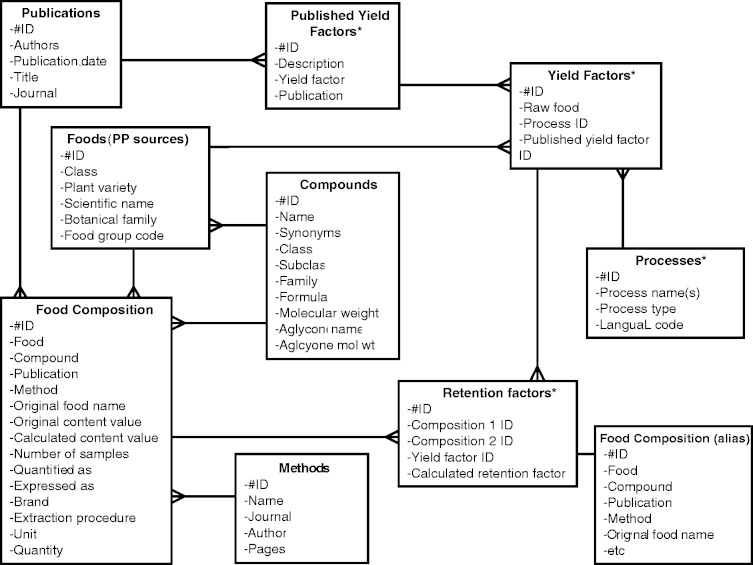
Entity-relationship diagram of the Phenol-Explorer 3.0 database showing new tables. New tables for the food processing module are denoted by asterisks.

Data were extracted from retained publications by researchers with expertise in food technology, nutrition or agriculture. Data from each publication were entered in a standardized sequence to ensure consistency. As with previous releases of Phenol-Explorer, data were entered into different tables of a Microsoft Access database. The first release of Phenol-Explorer stored data in five tables (4). Four of these were independent tables named *Foods*, *Publications*, *Compounds* and *Methods*, whose records were each given unique identifiers. A fifth table, *Food Composition*, listed each concentration value collected as a record, accompanied by codes linking to the food, publication, polyphenol quantified and method of analysis. To build the food processing module, data were entered into existing database tables as well as a further four tables that were added to the relational design: *Retention Factors*, *Published Yield Factors*, *Yield Factors* and *Processes* (Figure 1). The tables relevant to the retention factor module are described as follows:

*Food Composition*: Polyphenol contents of raw and processed foods were entered into this table and converted to mg/100 g or mg/100 ml in the same manner as for dedicated composition data. Data used for the new food processing update were not added to the part of the database dedicated to the polyphenol contents of foods.

*Retention Factors:* Identifiers for polyphenol contents of foods before and after processing were entered into this table along with an allocated yield factor for a given food and process. Retention factors, which describe the change in polyphenol content for a given food due to a given process were then calculated from the polyphenol contents of corresponding raw and processed foods and the yield factor value. Retention factors were stored in an additional field of this table.

*Published Yield Factors:* Yield factors describe the change in weight of a food due to processing. Each corresponds to an individual food and process. Original yield factors extracted from polyphenol literature were entered in this table, keeping exactly the description included in the original source (e.g. ‘potato, not peeled, microwaved’).

*Yield Factors:* Lists each combination of food and process for which retention factors are available and allocates to each a published yield factor, using the food and process descriptors of Phenol-Explorer (‘potato, raw’ and ‘microwaved’). Where an exact match was not available, the closest yield factor available was applied on a case-by-case basis (for instance, the published yield factor for ‘whole potato cooked by microwave’ was applied to ‘microwaved peeled potato’).

*Processes:* Lists the processes or combination of processes that have been applied to polyphenol-containing foods. Each process is allocated a unique identifier.

Retention factors stored in the retention factors table were aggregated to give a single value (average, minimum, maximum and standard deviation) for each individual food, process, polyphenol, analytical method (with or without hydrolysis) and measurement unit. In some cases, therefore, retention factors for different varieties of the same food (e.g. apple) have been aggregated, although retention factors for individual varieties are still available to view separately.

Further details about the methods used to build the Phenol-Explorer database can be found at http://www.phenol-explorer.eu/methods_used.

### Technical implementation of the update

The new Microsoft Access tables were exported to the MySQL database that feeds the web interface. For Phenol-Explorer 3.0, a faster, cleaner and more professional web interface has been implemented using the Bootstrap front-end framework (http://twitter.github.io/bootstrap/). This framework has also added support for mobile devices through the responsive design. The Ruby on Rails application framework (http://www.rubyonrails.org) has been upgraded for greater security and reliability and the javascript layer improved to allow faster page loading. The site is now integrated with MolDB, the same structure server used for the Human Metabolome database (http://www.hmdb.ca) (17), which allows the efficient calculation of structural properties (such as molecular weight, formula and physicochemical properties). The publicly accessible Phenol-Explorer 3.0 web server runs from an Apache 2 web server on a Debian Linux system.

### Querying and output

**Figure 2.**
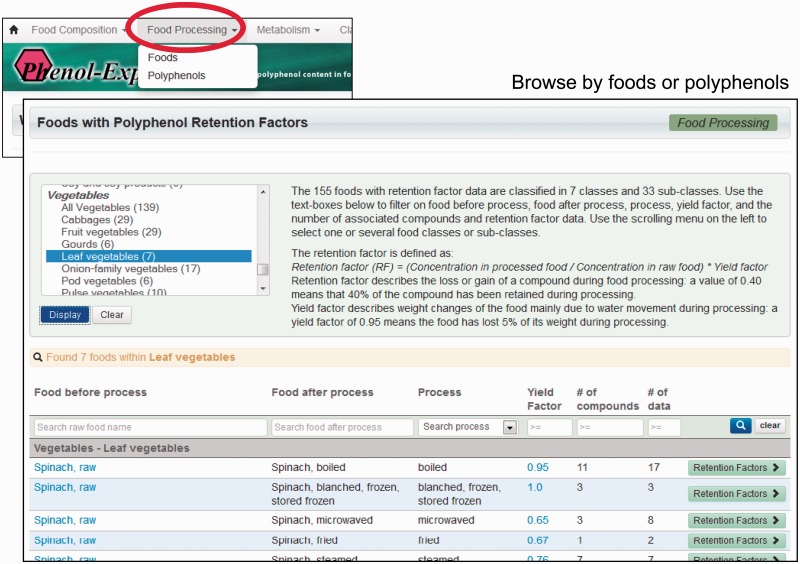
Integration of the new food processing module into the graphical user interface.

**Figure 3.**
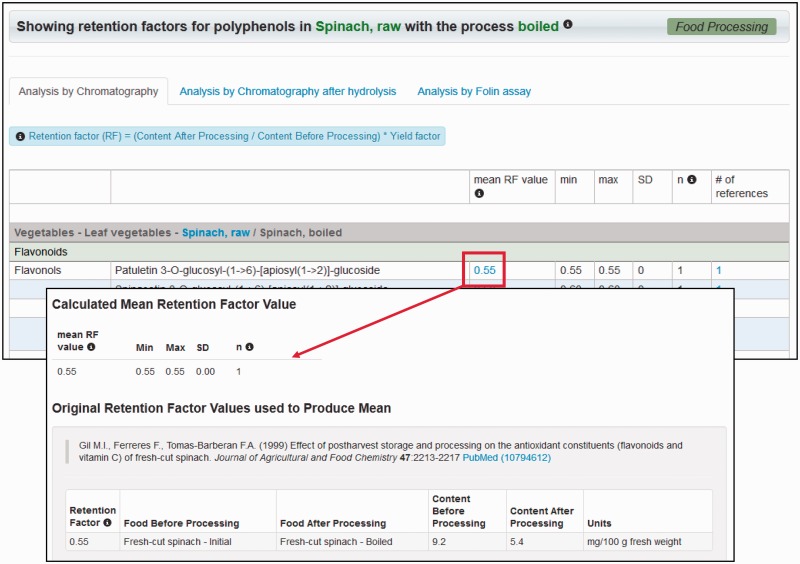
Food processing data output for spinach, raw to spinach, boiled. Searching or browsing for a food or polyphenol retrieves a list of aggregated retention factors whose components can then be viewed individually.

Retention factor data are searched for and retrieved in the same way as food composition data. A new button, *food processing*, is available in the site’s main toolbar, from which either all foods or all polyphenols with retention factors can be browsed. If foods are selected, a list of all food and process combinations, the yield factor used, number of polyphenols for which retention factors are available and volume of data is displayed. This list can be browsed and filtered by any field, as with metabolism and composition data (Figure 2). For each food and process combination, a summary of retention factors is available by clicking on the corresponding *retention factors* button. A new page then displays the mean aggregated retention factors for each polyphenol or polyphenol group, along with standard deviation, range and links to references. Clicking on aggregated retention factors themselves retrieves a summary of the individual retention factors that have been averaged, along with corresponding details and the original reference (Figure 3). Alternatively, *polyphenols* can be selected to list retention factors by polyphenol/process combination. Again, aggregated retention factors are viewed by clicking the *retention factors* button. Where aggregated retention factors are displayed, the value itself can be clicked to retrieve details of each individual retention factor used in the aggregation.

The simple search box in the top-right hand corner of any page on the site may also be used to search for food processing data. When a polyphenol or food is entered, a list of matching items is displayed in the form of boxes, which contain links to data. This display now contains the link *show retention factors*, which can be followed to retrieve aggregated retention factors. As with Phenol-Explorer composition and metabolism data, text boxes at the top of each column allow for further filtering of data.

For Phenol-Explorer 3.0, significant improvements to the overall user interface have been made. Pages for each of the three parts of the database (composition, metabolism and processing) have been more clearly separated and marked. The polyphenol and food classification hierarchy is displayed via a tree-like structure where the user can see previews of foods and polyphenols. The advanced search has been redesigned and the reports interface has been updated and improved.

## Discussion

For version 3.0 of the Phenol-Explorer database, we have added a new module on changes in the polyphenol contents of foods due to cooking and other processing methods. To our knowledge, it is the first database to collate this type of data for polyphenols. The USDA Table of Nutrient Retention Factors, first released in 1984, provides retention factors for some carotenoids, but not polyphenols. Phenol-Explorer is dedicated to this highly consumed group of bioactive phytochemicals. In addition, data are presented through a user-friendly web interface that allows rapid searches to be performed and the origins of data to be easily viewed.

In most cases, polyphenol contents of foods before and after processing were determined by high-performance liquid chromatography (HPLC) where identities could be verified with reference to authentic standards, and concentrations could be reliably calculated from chromatographic peak areas. The only exceptions to the use of this method was for determination of total anthocyanins, using the pH differential method, and for total polyphenols using the Folin-Ciocalteu method. The pH differential method calculates concentration of extracts based on the change in absorbance between two different pH values (18), while the Folin-Ciocalteu method uses a reagent that is reduced by phenolic groups to produce a measurable absorbance (19). Caution should be taken when comparing retention factors obtained by different methods, as polyphenols degraded during food cooking or processing may no longer be detectable by HPLC, but still retain an absorbance or capacity to reduce the Folin reagent.

**Figure 4.**
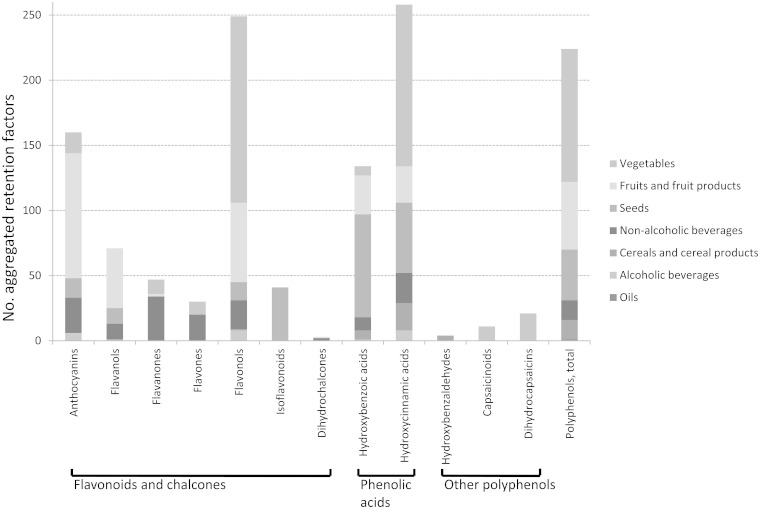
Volume of food processing data in Phenol-Explorer 3.0 by food group and polyphenol subclass. Each of the 1253 final retention factors represented is either a single retention factor obtained from the literature or two or more aggregated retention factors for one of 35 processes or combinations of processes.

Reliability of the averaged retention factor values depends on the number or raw and processed foods analyzed. For the Phenol-Explorer 3.0 Web site, as many values as possible have been averaged, where food, process, polyphenol and method are identical, resulting in a final count of 1253 retention factors derived from 4626 published retention factors (Figure 4). It should be noted that only one retention factor was available for some combinations of polyphenol, food and process, whereas many could be aggregated for others [e.g. 43 values for cyanidin 3-O-glucoside, pelargonidin 3-O-glucoside and pelargonidin 3-O-rutinoside in strawberry jam stored at room temperature (20)]. Aggregated retention factors, although sharing by definition a common food and processing method, may vary in other details, such as length of storage or plant variety. In some cases, plant variety may be responsible for greater variation in polyphenol content than processing method (21). Retention factor values should therefore be individually assessed according to user needs, taking into consideration the number and origin of aggregated values. Detailed information on samples and processes used to produce retention factors can be retrieved by clicking on the individual values.

For a substantial proportion of the data, polyphenol-containing food extracts were treated with acid or alkali to hydrolyze polyphenol glycosides and phenolic acid esters. Most polyphenols are found conjugated to monosaccharides or polyols in plant foods (22), and aglycones are usually found only in trace amounts unless the food has been fermented. This hydrolysis allowed different conjugated derivatives of the same parent compound to be grouped together and expressed as aglycone, where analytical sensitivity was not sufficient to detect or identify the individual forms or when appropriate standards for measuring conjugated polyphenols were not available. Therefore, retention factors calculated after such hydrolyses were treated separately and not aggregated with those obtained from untreated samples.

A retention factor is also a function of the yield factor, which accounts for change in weight of a food due to processing. Yield factors were not given in all publications, and in some cases, retention factor calculations were performed using a yield factor for a similar combination of food and process from another publication. This should be considered a limitation of the standardized and aggregated data. However, only yield factors were imputed, and the yield factor used is clearly displayed for each retention factor. Processing and cooking normally cause loss of nutrients, owing to degradation or oxidation caused by temperature or pH change. Therefore, retention factor values previously compiled for other nutrients were always <1 (in the USDA database for example, 100% retention or a value of 1 was assumed to be a maximum and any values in excess of this were rounded down to this figure). Although most polyphenols also degrade in this manner, retention factors >1, or an increase in the concentration of a particular polyphenol, are possible and occur in Phenol-Explorer 3.0. For example, aglycone concentrations may rise where processing causes polyphenol conjugates to lose their sugar moieties or where phenolic acids are hydrolyzed from their polyol esters. For example, a retention factor of 2.39 was calculated for quercetin aglycone in cauliflower following blanching (14). Where polyphenols are hydrolyzed before analysis, only a reduction in aglycone content is theoretically possible. In practice, the application of an unsuitable yield factor or poor analytical reproducibility could also explain an increase in polyphenol concentration. Care should therefore be taken when using retention factors greatly in excess of 1 for estimation of polyphenol intake.

Phenol-Explorer 1.0 was initially conceived to easily retrieve the polyphenol content of a food of interest. Although these estimations of polyphenol content were more advanced than what was previously available, they did not take into account the effects of cooking and storage on polyphenol-rich foods. In some cases, there were sufficient composition data on a particular processed food for a database entry separate from the raw food, such as orange juice produced from oranges. These processed foods were then fully documented in Phenol-Explorer 1.0. However, a paucity of data on polyphenol content for many other processed foods meant that they could not be included in the first release of the database. Release 3.0 allows the retrieval of the most suitable retention factor to be applied to more poorly documented processed foods. For those polyphenols for which more data were available, aggregated retention factors could produce more accurate values, although individual published retention factors are viewable if needed. One strength, therefore, of these food processing data is their variety and transparency and the resulting potential for imputation to missing foods, processes or polyphenols.

The data collated for Phenol-Explorer 3.0 allow the most detailed analysis so far of how different domestic and industrial processes affect the polyphenol contents of foods. The volume of data collected on different foods and processes corresponded with their importance in terms of polyphenol dietary exposure. For example, the fruit and vegetable food groups and their main polyphenols accounted for the majority of the data, and the flavonols and hydroxycinnamic acids were the most studied polyphenol subclasses (Figure 4). Domestic processes such as boiling, steaming, refrigeration and storage at room temperature were most commonly applied. The database also helps identify those areas where more study should be undertaken. Data are most notably lacking for the cereal and oil food groups, which may contribute a substantial proportion of polyphenols to some diets. Similarly, few data were available for canning and drying processes, which are applied industrially to many widely consumed polyphenol-rich foods. These aspects will be more extensively discussed in a separate article currently in preparation.

Phenol-Explorer is the first web database to include retention factors on this important class of dietary bioactives. As with previous releases, all data are traceable back to the original publications, and data will be periodically reviewed and new data added where possible, taking into account user comments. The database should be invaluable for the refinement of calculation of polyphenol content in processed foods and of dietary exposure to polyphenols in epidemiological studies. It will allow estimation of polyphenol intake to a greater degree of accuracy than ever before from dietary surveys, with a view to the long-term goal of clarifying the links between polyphenol intake and major chronic disease.

## Funding

Danone Research and the Institut National du Cancer, Paris (INCa 2011-105). Funding for open access charge: Institut National du Cancer

*Conflict of interest*. None declared.

## References

[1] Perez-Jimenez J, Neveu V, Vos F (2010). Systematic analysis of the content of 502 polyphenols in 452 foods and beverages: an application of the Phenol-Explorer database. J. Agric. Food Chem..

[2] Perez-Jimenez J, Fezeu L, Touvier M (2011). Dietary intake of 337 polyphenols in French adults. Am. J. Clin. Nutr..

[3] Ovaskainen M-L, Torronen R, Koponen JM (2008). Dietary intake and major food sources of polyphenols in Finnish adults. J. Nutr..

[4] Arts IC, Hollman PC (2005). Polyphenols and disease risk in epidemiologic studies. Am. J. Clin. Nutr..

[5] Scalbert A, Manach C, Morand C (2005). Dietary polyphenols and the prevention of diseases. Crit. Rev. Food Sci. Nutr..

[6] Hooper L, Kroon PA, Rimm EB (2008). Flavonoids, flavonoid-rich foods, and cardiovascular risk: a meta-analysis of randomized controlled trials. Am. J. Clin. Nutr..

[7] Hung HC, Joshipura KJ, Jiang R (2004). Fruit and vegetable intake and risk of major chronic disease. J. Natl. Cancer Inst..

[8] Arab L, Liu WQ, Elashoff D (2009). Green and black tea consumption and risk of stroke: a meta-analysis. Stroke.

[9] Peters U, Poole C, Arab L (2001). Does tea affect cardiovascular disease? A meta-analysis. Am. J. Epidemiol..

[10] van Dam RM, Feskens EJ (2002). Coffee consumption and risk of type 2 diabetes mellitus. Lancet.

[11] Miglio C, Chiavaro E, Visconti A (2008). Effects of different cooking methods on nutritional and physicochemical characteristics of selected vegetables. J. Agric. Food Chem..

[12] Bognár A, Piekarski J (2000). Guidelines for recipe information and calculation of nutrient composition of prepared foods (dishes). J. Food Comp. Anal..

[13] Haytowitz DB, Lemar LE, Pehrsson PR (2009). USDA's Nutrient Databank System—a tool for handling data from diverse sources. J. Food Comp. Anal..

[14] Bognar A (2002).

[15] Neveu V, Perez-Jimenez J, Vos F (2010). Phenol-Explorer: an online comprehensive database on polyphenol contents in foods. Database.

[16] Rothwell JA, Urpi-Sarda M, Boto-Ordonez M (2012). Phenol-Explorer 2.0: a major update of the Phenol-Explorer database integrating data on polyphenol metabolism and pharmacokinetics in humans and experimental animals. Database.

[17] Wishart DS, Jewison T, Guo AC (2013). HMDB 3.0: the Human Metabolome Database in 2013. Nucleic Acids Res..

[18] Lee J, Durst RW, Wrolstad RE (2005). Determination of total monomeric anthocyanin pigment content of fruit juices, beverages, natural colorants, and wines by the pH differential method: collaborative study. J. AOAC Int..

[19] Stratil P, Klejdus B, Kuban V (2006). Determination of total content of phenolic compounds and their antioxidant activity in vegetables—evaluation of spectrophotometric methods. J. Agric. Food Chem..

[20] Garcia-Viguera C, Zafrilla P, Romero F (1999). Color stability of strawberry jam as affected by cultivar and storage temperature. J. Food Sci..

[21] Amarowicz R, Carle R, Dongowski G (2008). Influence of postharvest processing and storage on the content of phenolic acids and flavonoids in foods. Mol. Nutr. Food Res..

[22] Crozier A, Jaganath IB, Clifford MN (2009). Dietary phenolics: chemistry, bioavailability and effects on health. Nat. Prod. Rep..

